# Assisting scalable diagnosis automatically via CT images in the combat against COVID-19

**DOI:** 10.1038/s41598-021-83424-5

**Published:** 2021-02-18

**Authors:** Bohan Liu, Pan Liu, Lutao Dai, Yanlin Yang, Peng Xie, Yiqing Tan, Jicheng Du, Wei Shan, Chenghui Zhao, Qin Zhong, Xixiang Lin, Xizhou Guan, Ning Xing, Yuhui Sun, Wenjun Wang, Zhibing Zhang, Xia Fu, Yanqing Fan, Meifang Li, Na Zhang, Lin Li, Yaou Liu, Lin Xu, Jingbo Du, Zhenhua Zhao, Xuelong Hu, Weipeng Fan, Rongpin Wang, Chongchong Wu, Yongkang Nie, Liuquan Cheng, Lin Ma, Zongren Li, Qian Jia, Minchao Liu, Huayuan Guo, Gao Huang, Haipeng Shen, Liang Zhang, Peifang Zhang, Gang Guo, Hao Li, Weimin An, Jianxin Zhou, Kunlun He

**Affiliations:** 1 Key Laboratory of Ministry of Industry and Information, Technology of Biomedical Engineering and Translational Medicine, Chinese PLA General Hospital, Beijing, 100853 People’s Republic of China; 2 Translational Medical Research Center, Chinese PLA General Hospital, Beijing, 100853 People’s Republic of China; 3 HKU Business School, The University of Hong Kong, Hong Kong, People’s Republic of China; 4 Department of Critical Care Medicine, Beijing Tiantan Hospital, Capital Medical University, Beijing, 100070 People’s Republic of China; 5 Department of Medical Imaging, Suizhou Hospital, Hubei University of Medicine (Suizhou Central Hospital), Suizhou, 431300 Hubei People’s Republic of China; 6 Department of Radiology, Wuhan Third Hospital, Tongren Hospital of Wuhan University, Wuhan, 430063 Hubei People’s Republic of China; 7 Department of Radiology, WenZhou Central Hospital, WenZhou, 325000 Zhejiang People’s Republic of China; 8 Department of Neurology, Beijing Tiantan Hospital, Capital Medical University, Beijing, 100070 People’s Republic of China; 9 Pulmonary and Critical Care Medicine, Chinese PLA General Hospital, Beijing, 100853 People’s Republic of China; 10 Department of Radiology, Chinese PLA General Hospital, Beijing, 100853 People’s Republic of China; 11 Department of Radiology, Xiantao First People’s Hospital, Affiliated to Yangtze University, Xiantao, 433000 Hubei People’s Republic of China; 12 Department of Radiology, The First People’s Hospital of Jiangxia District, Wuhan, 430200 Hubei People’s Republic of China; 13 Department of Radiology, Wuhan Jinyintan Hospital, Wuhan, 430040 Hubei People’s Republic of China; 14 Department of Medical Imaging, Affiliated Hospital of Putian University, Putian, 351100 Fujian People’s Republic of China; 15 Department of Radiology, Chengdu Public Health Clinical Medical Center, Chengdu, 610061 Sichuan People’s Republic of China; 16 Department of Radiology, Wuhan Huangpi People’s Hospital, Wuhan, 430300 Hubei People’s Republic of China; 17 Jianghan University Affiliated Huangpi People’s Hospital, Wuhan, 430300 Hubei People’s Republic of China; 18 Department of Radiology, Beijing Tiantan Hospital, Capital Medical University, Beijing, 100070 People’s Republic of China; 19 Department of Medical Imaging Center, Dazhou Central Hospital, Dazhou, 635000 Sichuan People’s Republic of China; 20 Department of Radiology, Beijing Daxing District People’s Hospital (Capital Medical University Daxing Teaching Hospital), Beijing, 100191 People’s Republic of China; 21 Department of Radiology, Shaoxing People’s Hospital (The First Affiliated Hospital of Shaoxing University), Shaoxing, 312000 Zhejiang People’s Republic of China; 22 Department of Radiology, The People’s Hospital of Zigui, Zigui, 443600 Hubei People’s Republic of China; 23 Department of Medical Imaging, Anshan Central Hospital, Anshan, 114001 Liaoning People’s Republic of China; 24 Department of Medical Imaging, Guizhou Provincial People’s Hospital, Guiyang, 550002 Guizhou People’s Republic of China; 25 Department of Computer Application and Management, Chinese PLA General Hospital, Beijing, 100070 People’s Republic of China; 26 Department of Automation, Tsinghua University, Beijing, 100084 People’s Republic of China; 27 China National Clinical Research Center for Neurological Diseases, Center for Bigdata Analytics and Artificial Intelligence, Beijing, 100070 People’s Republic of China; 28 Biomind Technology Co. Ltd, Beijing, 101300 People’s Republic of China; 29 Department of Radiology, 5th Medical Center, Chinese PLA General Hospital, Beijing, 100039 People’s Republic of China

**Keywords:** Viral infection, Computer science

## Abstract

The pandemic of Coronavirus Disease 2019 (COVID-19) is causing enormous loss of life globally. Prompt case identification is critical. The reference method is the real-time reverse transcription PCR (RT-PCR) assay, whose limitations may curb its prompt large-scale application. COVID-19 manifests with chest computed tomography (CT) abnormalities, some even before the onset of symptoms. We tested the hypothesis that the application of deep learning (DL) to 3D CT images could help identify COVID-19 infections. Using data from 920 COVID-19 and 1,073 non-COVID-19 pneumonia patients, we developed a modified DenseNet-264 model, COVIDNet, to classify CT images to either class. When tested on an independent set of 233 COVID-19 and 289 non-COVID-19 pneumonia patients, COVIDNet achieved an accuracy rate of 94.3% and an area under the curve of 0.98. As of March 23, 2020, the COVIDNet system had been used 11,966 times with a sensitivity of 91.12% and a specificity of 88.50% in six hospitals with PCR confirmation. Application of DL to CT images may improve both efficiency and capacity of case detection and long-term surveillance.

## Introduction

The world is suffering from the COVID-19 pandemic since its outbreak in December 2019^1–3^. COVID-19 is highly contagious and infected patients can be asymptomatic but infectious^4^. As of July 11, 2020, there have been over 12 million confirmed COVID-19 cases and 556,335 deaths worldwide^5^. Community transmission has been increasingly reported in more than 180 countries^5^. Before any effective and safe vaccine of COVID-19 becomes available in clinical settings, improving the efficiency of the current clinical pathways and the capacity of patient management are crucial to successfully combat the COVID-19 pandemic and possible resurgence in the future^6,7^. Case identification is an important first step for subsequent clinical triage and treatment optimization. The reference detection method is using the real-time reverse transcription PCR (RT-PCR) assay to detect viral RNA^1^. Several limitations of this assay may curb its prompt large-scale application^8–10^.

Chest computed tomography (CT) can effectively capture the manifestations of COVID-19 infections and even asymptomatic infections^10–12^. Deep learning, an artificial intelligence (AI) technology, has achieved impressive performance in the analysis of CT images^13–16^. Chest CT with the aid of deep learning offers promises to reduce the burden of prompt mass case detection, especially under the shortage of RT-PCR^17^. We developed an automated robust deep learning model, COVIDNet, by directly analyzing 3D CT images, to assist screening and diagnosis of COVID-19 infected patients. Furthermore, as of March 23, 2020, the COVIDNet system had been employed in 6 hospitals in China with PCR confirmation. We provided clinical insights into the image features extracted by COVIDNet and proposed a practical scenario on how the developed tool might improve clinical efficiency.

## Results

**Figure 1:**
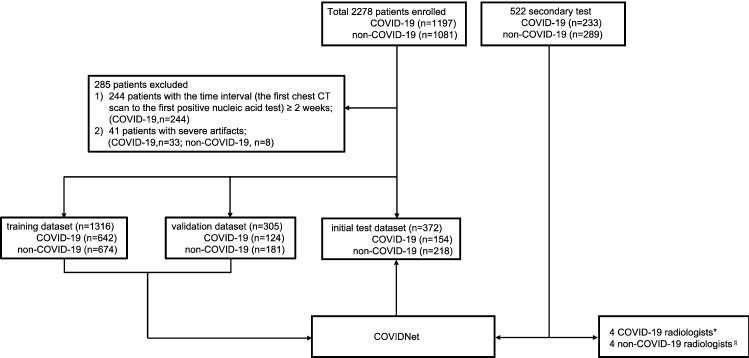
Flow diagram illustrating the division of model development data into training, validation, initial test, and secondary test on the secondary test dataset. The model development cohort consisted of 1197 COVID-19 patients and 1,081 non-COVID-19 patients. The secondary test cohort enrolled 233 COVID-19 patients and 289 non-COVID-19 patients. 285 patients were excluded due to ≥ 2 weeks interval of the first CT scan to the first positive nucleic acid test (244 patients), and severe artifacts (41 patients). The model development cohort was then divided into a training dataset (1316 patients), a validation dataset (305 patients), and an initial test dataset (372 patients) to train and fine-tune COVIDNet. A secondary test was then performed to test the model generalizability by comparing the diagnostic performance of COVIDNet and 8 different expert radiologists on the secondary test dataset. *COVID-19 radiologists: radiologists working at the COVID-19 designated hospitals; §non-COVID-19 radiologists: radiologists not working at the COVID-19 designated hospitals.

Two independent cohorts of 2,800 patients were retrospectively recruited for model development and secondary test (Fig. 1). The model development cohort enrolled 920 COVID-19 patients and 1,073 non-COVID-19 patients, and all the patients in this cohort were randomly divided into three non-overlapping sets at the patient level: training, validation, and initial test, approximately at a 3:1:1 ratio (Fig. 1, Supplementary Tables 1–4). The secondary test consisted of 233 COVID-19 patients and 289 non-COVID-19 pneumonia patients (Fig. 1, Supplementary Tables 5–7). Regarding the two cohorts, the training and validation datasets consisted of the images of all scans for each patient to train and fine-tune the COVIDNet system. However, the initial and the secondary test dataset only employed the first CT scan image of each patient to calculate the model performance at the first-diagnosis.

The initial test dataset included 372 patients, 41.4% (154/372) of which were confirmed COVID-19 cases. COVIDNet yielded a remarkable diagnostic performance, with an accuracy rate of 96.0% and an AUC of 0.986. More performance measures were shown in Table 1 and Extended Data Fig. 1. The model performance was based on the first CT scan of each patient. Note that our development dataset might have an overlap of 17 patients with the dataset of Li et al.^18^, including 14 patients in the training set, and 3 patients in the initial test set. Retraining and retesting without these patients yielded similar results (Supplementary Table 8 and Extended Data Fig. 2).

**Table 1:** COVIDNet diagnostic performance on the initial test dataset.

	COVID-19 (n = 154)	Non-COVID-19 (n = 218)
Accuracy	96.0% (93.8–97.8%)	
AUC	0.986 (0.972–0.996)	
Sensitivity	92.2% (87.7–96.2%)	98.6% (96.9–100.0%)
Specificity	98.6% (96.9–100.0%)	92.2% (87.7–96.2%)
PPV	97.9% (95.3–100.0%)	94.7% (91.6–97.4%)
NPV	94.7% (91.6–97.4%)	97.9% (95.3–100.0%)
F1 score	95.0% (92.2–97.4%)	96.6% (94.8–98.2%)

372 patients were considered, including 154 COVID-19 patients and 218 non-COVID-19 patients. The diagnostic metrics of accuracy, AUC, sensitivity, specificity, PPV, NPV, and F1 score were calculated as well as the corresponding CIs. *AUC* area under the ROC curve, *PPV* positive predictive value, *NPV* negative predictive value, *CI* confidential interval.

In the secondary test, COVIDNet significantly outperformed all eight radiologists, which correctly diagnosed 492 of 522 patients, with an accuracy of 94.3% (CI 92.1%-96.1%) and an AUC of 0.981 (CI 0.969–0.990) (Table 2). Cohen’s κ coefficient^19^ was used to access inter-rater agreement between COVIDNet and the three radiologist groups (all radiologists, the radiologists from the COVID-19 designated hospitals, and the other radiologists) (Supplementary Table 9). Median inter-rater agreement among the radiologists in each group was good, with κ = 0.658 [IQR 0.539–0.776], κ = 0.738 [IQR 0.680–0.797], and κ = 0.600 [IQR 0.502–0.699], respectively. The agreement was good between each group of radiologists and our model (κ = 0.745, 0.746, and 0.678, respectively). The agreement between the COVID-19 radiologists and the non-COVID-19 radiologists was excellent (κ = 0.853).

**Table 2:** Diagnostic performance for each patient among COVIDNet and eight radiologists on the secondary test cohort.

	Accuracy	Sensitivity	Specificity	PPV	NPV	F1-score
COVIDNet	94.3% (92.1–96.1%)	93.1% (89.7–96.2%)	95.1% (92.5–97.5%)	93.9% (90.6–96.8%)	94.5% (91.7–96.9%)	93.5% (91.1–95.7%)
COVID-19 radiologists*						
Radiologist A (6 year)	82.1%	76.4%	86.8%	82.4%	82.0%	79.2%
Radiologist B (8 year)	85.1%	80.7%	88.6%	85.1%	85.1%	82.8%
Radiologist C (13 year)	83.3%	73.8%	91.0%	86.8%	81.2%	79.7%
Radiologist D (18 year)	85.2%	75.1%	93.4%	90.2%	82.3%	81.9%
Average	83.9%	76.5%	90.0%	86.1%	79.7%	80.9%
**non-COVID-19 radiologists**^§^						
Radiologist E (10 year)	78.4%	82.4%	75.1%	72.7%	84.1%	77.2%
Radiologist F (11 year)	81.0%	64.8%	94.1%	89.9%	76.9%	75.3%
Radiologist G (21 year)	72.4%	45.5%	94.1%	86.2%	68.2%	59.5%
Radiologist H (23 year)	83.9%	79.9%	87.2%	83.4%	84.3%	81.6%
Average	78.9%	68.2%	87.6%	83.1%	78.4%	73.4%

Four radiologists were from COVID-19 designated hospitals and the others were not. For COVID-19 radiologists, radiologist A-D had chest CT image trained experience of 6, 8, 13, and 18 years, respectively. Meanwhile, for the non-COVID-19 radiologist, radiologist E–H had 10, 11, 21, and 23 years of experience, respectively. *PPV* positive predictive value, *NPV* negative predictive value. *COVID-19 radiologists: radiologists working at the COVID-19 designated hospitals; ^§^**non-COVID-19 radiologists**: radiologists not working at the COVID-19 designated hospitals.

**Figure 2:**
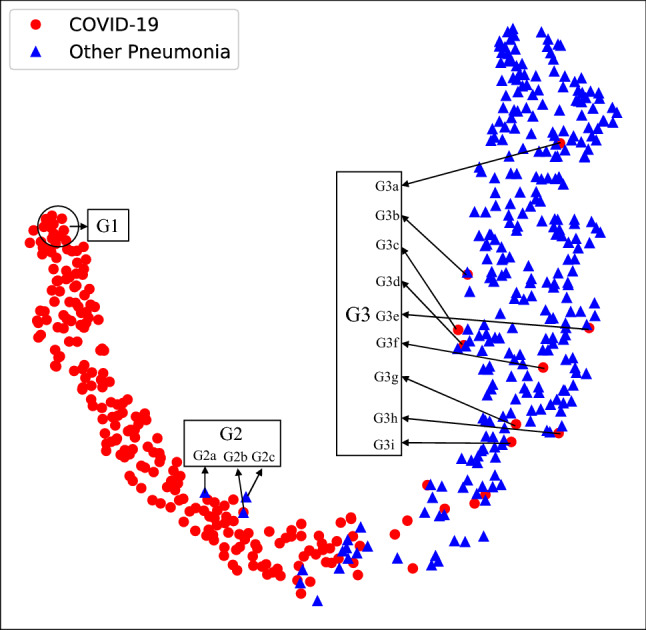
t-SNE of CT images of COVID-19 and other causes of pneumonia on the secondary test dataset. G1 depicted chest CT images of COVID-19 pneumonia with the highest differentiation. G2 represented three cases of false-positive prediction of COVIDNet. G3 pointed out nine cases of false-negative prediction of COVIDNet. CT image manifestations of G1 to G3 were illustrated in Supplementary Table 10.

The t-SNE representation of chest CT showed two clear clusters, color-coded by the class labels (Fig. 2). Most cases are located within their respective clusters, suggesting that COVIDNet successfully extracted distinct CT features of COVID-19 pneumonia. We selected three groups of representative cases (G1, G2, G3 in Fig. 2), and presented their CT manifestations along with the probability of COVID-19 in Extended Data Table 8. A typical manifestation of COVID-19 pneumonia is multiple ground-glass opacity (GGO) in the subpleural area of bilateral lungs. Radiologists confirmed a similar manifestation in the COVID-19 cluster (for example, the G1 red points). As for the misclassified COVID-19 cases (the G3 red points), three cases had no definite finding, which was difficult to identify only using the images; the other cases had extensive GGO with partial consolidation or combined with pleural effusion and interstitial edema, which were not the typical manifestations of COVID-19. The misclassified non-COVID-19 cases (the G2 blue points) consisted of one bacterial and two influenza B patients, which were classified as COVID-19 due to the appearance of extensive GGO.

**Figure 3:**
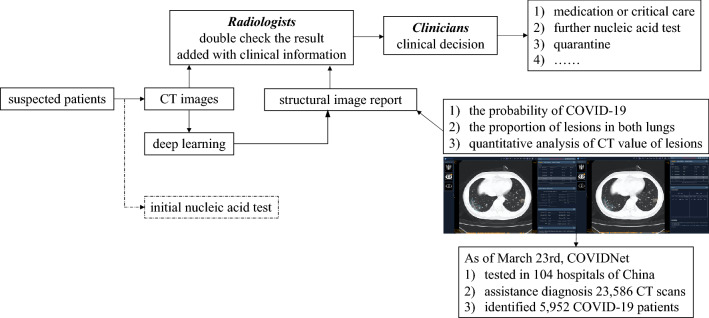
The pipeline of COVIDNet application in the real world.

**Figure 4:**
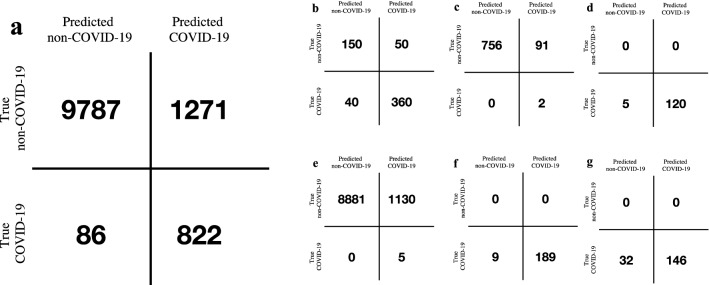
The performance of COVIDNet in the real-world application. (**a**) The confusion matrix of COVIDNet in the 6 hospitals, (**b**–**g**) the confusion matrix of COVIDNet in each hospital (**b**–**g** represents Hospital 1–6, respectively).

After the secondary test, we deployed the COVIDNet system in 6 hospitals to assist radiologists to screen suspected patients upon initial contact. The application pipeline of COVIDNet was illustrated in Fig. 3 and Extended Data Fig. 3. The pipeline aided efficiency improvement of the clinical pathway, which offered generalizable clinical insights for regions under considerable strains of nucleic test kits, with limited testing facilities, or facing community transmission epidemic. As of March 23, 2020, the COVIDNet system had been used to process 11,966 CT scans in six hospitals with PCR confirmation, resulting in a sensitivity of 90.52% and a specificity of 88.50% (Fig. 4 and Supplementary Table 11).

## Discussion

The COVID-19 pandemic continues to spread widely around the world. Until an effective vaccine becomes available in clinical use, we are in the combat against SARS-CoV-2 for the foreseeable near future. Accurate and prompt diagnosis of COVID-19 infection is essential for patient management. The specified criteria described in the current COVID-19 clinical management guideline have faced several challenges^20^. As the primary diagnostic tool, the nucleic acid test has several disadvantages^21^. Early clinical manifestations of COVID-19 are fever, cough, and dyspnea that are similar to non-COVID-19 viral pneumonia. Although chest radiograph is the initial screening image tool in some countries around the world, chest CT has been a vital part of the COVID-19 infection diagnostic pathway. COVID-19 infection with the main CT presentation of GGO can be easily confused with other viral pneumonia and fungal pneumonia. COVID-19 infection with the main CT manifestation of consolidation may be confused with a bacterial infection.

Our research showed that COVIDNet offered one powerful tool for screening the COVID-19 suspected patients. It could distinguish COVID-19 from other pneumonia infections promptly and accurately. The secondary test showed COVIDNet’s robustness against seven other types of pneumonia with confirmed pathogen evidence and various CT devices, as well as its faster and more accurate performance over expert radiologists. Our results also showed that the radiologists from the COVID-19 designated hospitals performed better than those from the non-epidemic regions. The excellent inter-rater reliability among the radiologists, together with their overall poorer performance against COVIDNet, suggested that COVIDNet provided more unbiased results and captured clinically important features of COVID-19 infections that might not have been detected by the human experts, given the fact that all COVID-19 cases were confirmed via nucleic test.

One recent study developed a deep learning screening tool for COVID-19^18^. They extracted features from each axial CT scan of a patient independently and aggregated the stack of features right before making the classification decision. On the contrary, our COVIDNet model directly extracted spatial features from the entire 3D CT scan using a true three-dimensional deep learning model. We also demonstrated the generalizability of our model on a secondary test dataset through comparison with expert radiologists. Most importantly, COVIDNet had been deployed for clinical use in 6 hospitals in China with PCR confirmation as of March 23rd, 2020.

Lacking methods for visualizing how deep learning works has been one of the major bottlenecks for its application in medical settings. To further investigate how COVIDNet made classification decisions, we visualized the extracted features using t-Distributed Stochastic Neighbor Embedding (t-SNE)^22^. The results showed that COVIDNet indeed extracted image features that could separate COVID-19 from the other types of pneumonia. We reported image signatures from representatives of the correctly classified COVID-19 cases, the misclassified COVID-19, and non-COVID-19 cases. Such image signatures could offer useful insights for clinical decisions. However, due to the limitations of the indistinct outline of lesion regions in the CT images, it would be subjective to classify COVID-19 and non-COVID-19 pneumonia by the approach of image labeling and segmentation, which is a traditional pathway to illustrate the difference of the diseases.

When facing an outbreak of COVID-19, often with a severe shortage of medical personnel, prompt and accurate image review and interpretation might be a key limiting factor for appropriate clinical decision making. COVIDNet can rapidly detect clinically relevant lung lesions from hundreds of CT images. Together with the probability sorting, COVIDNet may greatly improve the screening and diagnosis efficiency. Besides, COVIDNet can automatically quantify the proportion of image abnormalities, supporting further clinical decisions. However, CT presentations for patients with COVID-19 vary dramatically according to stages of the disease, especially for those with basic diseases and complications. Other types of pneumonia may also share image abnormalities with COVID-19. Deep learning technology may not perform well under these circumstances. Therefore, a patient’s epidemiological and clinical information needs to be closely integrated for further clinical decision-making in the diagnosis and treatment of COVID-19, and the scores produced by the model are not calibrated. Therefore, even though they can serve as a proxy for the classification confidence, their interpretation is rooted in accumulated experience obtained by integrating the model with clinical practice. Moreover, the slight overlap in hospitals between the model development cohort and the external validation may lead to the imperfection of the external validation. Above all, no one can ignore that this virus is evolving in directions that we don’t know yet^23^. In this particular occasion, COVIDNet would serve as an effective tool for routine screening in clinical settings where chest CT is prescribed. The screening role of COVIDNet may be limited in regions where chest radiography is the primary investigation method instead of CT.

In conclusion, we have developed an automated classification neural network model, COVIDNet, specifically designed to distinguish COVID-19 from seven other types of pneumonia with confirmed pathogens through analyzing patients’ 3D chest CT scans. In principle, the model can be deployed anywhere in the world with CT imaging capability at a low cost and provide radiological decision support where COVID-19 imaging diagnosis expertise is scarce, especially when facing COVID-19 outbreaks. Our results warrant further validation in future studies.

## Methods

### Datasets

We retrospectively recruited two cohorts for model development and secondary test, with a total of 2,800 patients (1,430 COVID-19 patients and 1,370 non-COVID-19 patients). And only the non-contrast scans were enrolled in this study. The model development dataset consisted of CT scans from 2,278 pneumonia patients, who suffered from either COVID-19 or other types of pneumonia. We collected 1,197 COVID-19 cases between January 5, 2020 and March 1, 2020 from ten designated COVID-19 hospitals in China (Supplementary Table 1). These COVID-19 cases were confirmed by positive results from RT-PCR assays testing nasal or pharyngeal swab specimens. We also randomly selected 1,081 non-COVID-19 patients with chest CT abnormalities according to the criteria listed in Supplementary Table 2 from patients that were hospitalized between November 18, 2018 and February 21, 2020 in three other general hospitals in China (Supplementary Table 4).

We excluded 285 patients under the following two circumstances after screening all images by two senior radiologists with 30-year work experience (Fig. 1): 244 COVID-19 patients with the time between the first CT scan and the first positive nucleic acid test longer than two weeks; and 41 patients with large breathing or body motion artifacts, including 33 COVID-19 patients and 8 non-COVID-19 patients.

For the secondary test cohort, we collected 233 COVID-19 cases between March 2, 2020 to March 13, 2020 from four COVID-19 designated hospitals. We also randomly selected 289 patients that were hospitalized between February 22, 2020 and March 1, 2020 in two general hospitals in China. Two of the COVID-19 hospitals and one non-COVID-19 hospital are also enrolled in the model development cohort. The inclusion criteria of COVID-19 and non-COVID-19 patients are the same as described above.

All CT scans of the two cohorts were performed upon the first contact, with patients in the supine position at full inspiration, and covered the whole chest.

### Study ethics

This study is compliant with the “Guidance of the Ministry and Technology (MOST) for the Review and Approval of Human Genetic Resources”. All the CT image data were obtained from Chinese PLA General Hospital, Suizhou Central Hospital, Wuhan Third Hospital, Wenzhou Central Hospital, Xiantao First People’s Hospital affiliated to Yangtze University, The First People's Hospital of Jiangxia District, Wuhan Jinyintan Hospital, Affiliated Hospital of Putian University, Chengdu Public Health Clinical Medical Center, Wuhan Huangpi People's Hospital, Dazhou Central Hospital, Beijing Daxing District People's Hospital, Shaoxing People's Hospital, The People’s Hospital of Zigui, Anshan Central Hospital, Guizhou Provincial People’s Hospital, 5th Medical Center of Chinese PLA General Hospital. This study was approved by the Ethics Committee (EC) of all the hospitals, and the written informed consent was waived by the ECs of all the hospitals since the data under evaluation has been de-identified and the research poses no potential risk to patients. The study follows the Declaration of Helsinki. The Trial Registration Number is ChiCTR2000030390 in Chinese Clinical Trial Registry, http://www.chictr.org.cn/showproj.aspx?proj=50224.

### CT image collection and preprocessing

CT images were obtained from different scanners at multiple imaging centers. The detailed CT scan setting and device distribution were listed in Supplementary Table 12 and Extended Fig. 4. COVIDNet was a classification neural network that classifies 3D CT images to either the COVID-19 pneumonia class or the non-COVID-19 pneumonia class. CT scans from different sources had various numbers of slices and slice dimensions. For unification, each of the 3D CT scan volumes was preprocessed in the following way. We first removed extreme voxel intensities by clipping those outside the range [− 1024, 1024] to the interval edges, and linearly scaled the clipped intensities to [0,1]. We subsequently resized the 3D scans to a stack of 64 square axial images of dimension 512 through linear interpolation and cropped the central square region of size 384 on each 2D axial slice, yielding a stack of 64 axial images of size 384, which was the input to the model. Preprocessed image examples were included in Extended Fig. 5.

### Architecture of COVIDNet

The structure of COVIDNet is illustrated in Extended Fig. 6, which is a modified DenseNet-264 model consisting of 4 dense blocks^24^. Each dense block has different numbers of composition units. Each unit consists of two sequentially connected stacks with an instance normalization layer^25^, a ReLU activation layer, and a convolution layer. It receives feature maps from all preceding units in the same dense block through dense connections. The training batch size is 8. We adopted Adam optimizer^26^ with a learning rate of 0.001 to minimize the binary cross-entropy loss. The model was developed using TensorFlow (version 1.8 with CUDA V9.1.85 and cuDNN 7.0.5) on 16 T P100 GPU.

### Model evaluation

The performance of the model was evaluated using accuracy, sensitivity, specificity, positive predictive value (PPV), negative predictive value (NPV), and F1 score. The receiver operating characteristic (ROC) curve and confusion matrix were generated based on the classification results. The area under the ROC (AUC) was also calculated. Bootstrap with 10,000 replications was used to calculate the 95% confidence interval for each metric. Evaluation results were obtained and visualized using python libraries, including NumPy (v.1.16.4), pandas (v.0.25.3), scikit-learn (v.0.19.2), and Matplotlib (v.2.1.2).

Furthermore, the performance of COVIDNet was compared with eight independent expert radiologists with 6–23 years of experience, on the diagnosis of COVID-19 using the secondary test dataset. Four radiologists are from the COVID-19 designated hospitals and the other four are not. To ensure that the radiologists could concentrate on the trail, each of them only read CT images no more than two hours per day under the surveillance of one research assistant. Before the radiologists initiated the CT image reading, the research assistant informed each radiologist about the CT signs in the guidelines to eliminate knowledge bias. The true pneumonia class was blinded to all the radiologists. We also used Cohen’s $\kappa$ coefficient to evaluate the inter-rater agreement among COVIDNet and the eight radiologists (Supplementary Table 9)^19^. We categorized κ coefficients as follows: poor (0 < κ ≤ 0.20), fair (0.20 < κ ≤ 0.40), moderate (0.40 < κ ≤ 0.60), good (0.60 < κ ≤ 0.80), and excellent (0.80 < κ ≤ 1.00).

To further understand the model’s classification decision, we visualized the extracted feature distribution of the model using t-Distributed Stochastic Neighbor Embedding (t-SNE)^22^, an unsupervised non-linear dimension reduction algorithm commonly used to visualize high dimensional data. It projects high-dimensional feature maps right before the final fully-connected layer of COVIDNet onto a two-dimensional space and converts similarities between the original data pairs to similarities between the projected data pairs in the two-dimensional space. Since it considers the local structure so that after projection, it can reveal interesting clusters among the data.

## Supplementary Information

**Supplementary Information.**

## Data Availability

We provide free access to the code at the following link: https://github.com/WingsOfPanda/covid-19-project. The CT image data and the clinical data will be available only for research purposes after obtaining the agreement of the corresponding authors.
