# Supplementary material for: Assisting scalable diagnosis automatically via CT images in the combat against COVID-19
Source: Sci Rep. 2021 Feb 18;11:4145. doi: 10.1038/s41598-021-83424-5 (PMC7892869; doi:10.1038/s41598-021-83424-5)
Supplement: Supplementary file 1 — Supplementary Information. [file 41598_2021_83424_MOESM1_ESM.docx]

# Supplementary Material

**Title: Assisting Scalable Diagnosis Automatically via CT Images in the Combat against COVID-19**

Bohan Liu^1,2#^, Pan Liu^1,2#^, Lutao Dai^3#^, Yanlin Yang^4#^, Peng Xie^5#^, Yiqing Tan^6#^, Jicheng Du^7#^, Wei Shan^8#^, Chenghui Zhao^1,2^, Qin Zhong^1,2^, Xixiang Lin^1,2^, Xizhou Guan^9^, Ning Xing^10^, Yuhui Sun^1,2^, Wenjun Wang^1,2^, Zhibing Zhang^11^, Xia Fu ^12^, Yanqing Fan^13^, Meifang Li^14^, Na Zhang^15^, Lin Li^16,17^, Yaou Liu^18^, Lin Xu^19^, Jingbo Du^20^, Zhenhua Zhao^21^, Xuelong Hu^22^, Weipeng Fan^23^, Rongpin Wang^24^, Chongchong Wu^10^, Yongkang Nie^10^, Liuquan Cheng^10^, Lin Ma^10^, Zongren Li^1,2^, Qian Jia^1,2^, Minchao Liu ^25^, Huayuan Guo ^25^, Gao Huang^26^, Haipeng Shen^3,27^, Liang Zhang^28^, Peifang Zhang^28^, Gang Guo^28^, Hao Li^27^, Weimin An^29*^, Jianxin Zhou^4*^, Kunlun He^1,2*^

1. Key Laboratory of Ministry of Industry and Information Technology of Biomedical Engineering and Translational Medicine, Chinese PLA General Hospital, Beijing, 100853, P.R.China
2. Translational Medical Research Center, Chinese PLA General Hospital, Beijing, 100853, P.R.China
3. HKU Business School, The University of Hong Kong, Hong Kong, P.R.China
4. Department of Critical Care Medicine, Beijing Tiantan Hospital, Capital Medical University, Beijing, 100070, P.R.China
5. Department of Medical Imaging, Suizhou Hospital, Hubei University of Medicine (Suizhou Central Hospital), Suizhou, Hubei, 431300, P.R.China.
6. Department of Radiology, Wuhan Third Hospital, Tongren Hospital of Wuhan University, Wuhan, Hubei, 430063, P.R.China
7. Department of Radiology, WenZhou Central Hospital, WenZhou, Zhejiang, 325000, P.R.China
8. Department of Neurology, Beijing Tiantan Hospital, Capital Medical University, Beijing, 100070, P.R.China
9. Pulmonary and Critical Care Medicine, Chinese PLA General Hospital, Beijing, 100853, P.R.China
10. Department of Radiology, Chinese PLA General Hospital, Beijing, 100853, P.R.China
11. Department of Radiology, Xiantao First People's Hospital affiliated to Yangtze University, Xiantao, Hubei, 433000, P.R.China
12. Department of Radiology, The First People's Hospital of Jiangxia District, Wuhan, Hubei,430200, P.R.China
13. Department of Radiology, Wuhan Jinyintan Hospital, Wuhan, Hubei, 430040, P.R.China
14. Department of Medical Imaging, Affiliated Hospital of Putian University, Putian, Fujian, 351100, P.R.China
15. Department of Radiology, Chengdu Public Health Clinical Medical Center, Chengdu, Sichuan, 610061, P.R.China
16. Department of Radiology, Wuhan Huangpi People's Hospital, Wuhan, Hubei, 430300, P.R.China
17. Jianghan University Affiliated Huangpi People’s Hospital, Wuhan, Hubei, 430300, P.R.China
18. Department of Radiology, Beijing Tiantan Hospital, Capital Medical University, Beijing, 100070, P.R.China
19. Department of Medical Imaging Center, Dazhou Central Hospital, Dazhou, Sichuan, 635000, P.R.China
20. Department of Radiology, Beijing Daxing District People's Hospital (Capital Medical University Daxing Teaching Hospital), Beijing, 100191, P.R.China
21. Department of Radiology, Shaoxing People's Hospital (The First Affiliated Hospital of Shaoxing University), Shaoxing, Zhejiang, 312000, P.R.China
22. Department of Radiology, The People’s Hospital of Zigui, Zigui, Hubei, 443600, P.R.China
23. Department of Medical Imaging, Anshan Central Hospital, Anshan, Liaoning, 114001, P.R.China
24. Department of Medical Imaging, Guizhou Provincial People’s Hospital, Guiyang, Guizhou, 550002, P.R.China
25. Department of Computer Application and Management, Chinese PLA General Hospital，Beijing, 100070, P.R.China
26. Department of automation, Tsinghua University, Beijing, 100084, P.R.China
27. China National Clinical Research Center for Neurological Diseases, Center for Bigdata Analytics and Artificial Intelligence, Beijing, 100070, P.R.China
28. Biomind Technology Co. Ltd, Beijing, 101300, P.R.China
29. Department of Radiology, 5th Medical Center, Chinese PLA General Hospital, Beijing, 100039, P.R.China

^#^These authors contributed equally to this work.

Bohan Liu, Pan Liu, Lutao Dai, Wei Shan, Peng Xie, Yiqing Tan, Jicheng Du, Yanlin Yang

^*^Correspondence:

Kunlun He ([kunlunhe@plagh.org](mailto:kunlunhe@plagh.org)), M.D, Ph.D. Translational Medicine Research Center, Chinese PLA General Hospital; Key Laboratory of Ministry of Industry and Information Technology of Biomedical Engineering and Translational Medicine, Chinese PLA General Hospital, Beijing, 100853, P.R.China.

Jianxin Zhou ([zhoujx.cn@icloud.com](mailto:zhoujx.cn@icloud.com)), MD, PhD. Department of Critical Care Medicine, Beijing Tiantan Hospital, Capital Medical University, Beijing, 100070, P.R.China.

Weimin An ([anweimin3021966@163.com](http://anweimin3021966@163.com)), MD, PhD. Department of Radiology, 5th Medical Center, PLA General Hospital, Beijing, 100039, P.R.China.

**Extended Data 1. Additional Information on the Model Development Set**

|  | | number of patients | | | number of scans | | |
| --- | --- | --- | --- | --- | --- | --- | --- |
|  |  | **Training** | **Validation** | **Initial test** | **Training** | **Validation** | **Initial test** |
| COVID-19 Designated Hospital | Suizhou Hospital | 137 | 33 | 48 | 373 | 95 | 127 |
|  | Xiantao First People's Hospital affiliated to Yangtze University | 223 | 48 | 51 | 126 | 25 | 17 |
|  | The First People's Hospital of Jiangxia District | 32 | 5 | 3 | 126 | 25 | 17 |
|  | Wuhan Third Hospital | 54 | 7 | 4 | 93 | 13 | 7 |
|  | WenZhou Central Hospital | 100 | 22 | 24 | 231 | 49 | 67 |
|  | Wuhan Jinyintan Hospital | 26 | 9 | 11 | 46 | 15 | 16 |
|  | The People’s Hospital of Zigui | 16 | 0 | 3 | 74 | 0 | 12 |
|  | Affiliated Hospital of Putian University | 27 | 0 | 4 | 47 | 0 | 7 |
|  | Wenzhou Second Hospital | 13 | 0 | 3 | 46 | 0 | 4 |
|  | Wuhan Huangpi People's Hospital | 14 | 0 | 3 | 18 | 0 | 5 |
|  | In total | 642 | 124 | 154 | 1305 | 246 | 313 |
| Non-COVID-19 Designated Hospital | The first medical center, Chinese PLA General Hospital | 452 | 129 | 148 | 1045 | 261 | 320 |
|  | The Second Affiliated Hospital of Xiamen Medical College | 164 | 36 | 55 | 427 | 99 | 143 |
|  | Beijing Tiantan Hospital | 58 | 16 | 15 | 58 | 18 | 16 |
|  | In total | 674 | 181 | 218 | 1530 | 378 | 479 |

**Supplementary** **Table 1. The number of patients and scans in each enrolled hospital in the model development cohort.**

| **ICD-10 code** | **Definition** |
| --- | --- |
| J10·0 | Influenzal (broncho) pneumonia, influenza virus identified |
| J12·0 | Adenoviral pneumonia |
| J15·0 | Pneumonia due to Klebsiella pneumonia |
| J15·1 | Pneumonia due to Pseudomonas |
| J15·2 | Pneumonia due to staphylococcus |
| J15·3 | Pneumonia due to streptococcus, group B |
| J15·5 | Pneumonia due to Escherichia coli |
| J15·7 | Pneumonia due to Mycoplasma pneumoniae |
| B25·0 | Pneumonia in cytomegalovirus diseases |
| B44·0-B44·1 | Pneumonia in aspergillosis |

**Supplementary** **Table 2. Inclusion criteria of non-COVID-19 in model development cohort were based on the ICD-10 code with 10 items.**

|  | **COVID-19 (n=920)** | **non-COVID-19 (n=1,073)** |
| --- | --- | --- |
| **Gender**  **Male**  **Female** | 900/920 (97.8%)  498/900 (55.3%)  402/900 (44.7%) | 1,063/1,073 (99.1%)  613/1063 (57.7%)  450/1063 (42.3%) |
| **Age** | 902/920 (98.0%)  49.7±15.2 [48.8-50.6] | 1055/1073 (98.3%)  54.7±18.5 [53.8-55.6] |

**Supplementary** **Table 3. Demographic characteristics of the model development dataset.**

| **Training Set** | | | **Validation Set** | | | **Initial Test Set** | | |
| --- | --- | --- | --- | --- | --- | --- | --- | --- |
|  | number of patients | |  | number of patients | |  | number of patients | |
| number of scans | COVID-19 | non-COVID-19 | number of scans | COVID-19 | non-COVID-19 | number of scans | COVID-19 | non-COVID-19 |
| 1 | 318 | 318 | 1 | 68 | 81 | 1 | 97 | 75 |
| 2 | 153 | 190 | 2 | 20 | 51 | 2 | 63 | 32 |
| 3 | 77 | 58 | 3 | 15 | 26 | 3 | 29 | 26 |
| 4 | 54 | 43 | 4 | 14 | 10 | 4 | 14 | 13 |
| 5 | 25 | 24 | 5 | 5 | 5 | 5 | 3 | 5 |
| 6 | 8 | 9 | 6 | 2 | 4 | 6 | 3 | 2 |
| ≥7 | 7 | 32 | ≥7 | 0 | 4 | ≥7 | 9 | 1 |

**Supplementary** **Table 4. The number of patients with different times (1-≥7) of CT scan in training, validation set and initial test dataset. The detailed numbers of COVID-19 and non-COVID-19 patients were listed for each dataset.** The training and validation datasets consisted of the images of all scans for each patient, however, the initial test dataset only employed the first CT scan image of each patient.

**Extended Data 2. Additional Information on the Secondary Test Dataset**

|  | | number of patients | number of scans |
| --- | --- | --- | --- |
| COVID-19 Designated Hospital | Suizhou Hospital | 139 | 139 |
|  | Wuhan Third Hospital | 22 | 22 |
|  | Shaoxing People's Hospital | 18 | 18 |
|  | The fifth medical center, Chinese PLA General Hospital | 54 | 54 |
|  | In total | 233 | 233 |
| Non-COVID-19 Designated Hospital | The first medical center, Chinese PLA General Hospital | 69 | 69 |
|  | The fifth medical center, Chinese PLA General Hospital | 220 | 220 |
|  | In total | 289 | 289 |

**Supplementary** **Table 5. The number of patients and scans in each enrolled hospital in the secondary test cohort.**

| **CT findings** | **new or progressive pulmonary infiltrates, consolidation, ground-glass opacities, or interstitial changes** |
| --- | --- |
| etiological or serological evidence | 1) influenza A and B  the nucleic acid amplification test from throat swabs  2) cytomegalovirus infection  the nucleic acid quantification in peripheral blood  3) adenovirus infection  the clinical diagnosis and the positive serum antibody IgM in acute  4) mycoplasma infection  the serum antibody index, a four-fold rise in acute to convalescent  5) bacterial infection  the identification of certain pathogens from the carefully obtained microbiological evidence, such as sputum and lower respiratory tract specimens.  6) fungus infection  the presence of a host factor, a clinical feature and mycologic evidence |

**Supplementary** **Table 6. The inclusion criteria of non-COVID-19 patients in the external validation cohort. Each patient has to yield both CT findings and the corresponding etiological or serological.**

|  | **Age** | **Gender** | |
| --- | --- | --- | --- |
|  |  | **male** | **female** |
| COVID-19 (n=233) | 50·0(47·0-53·0) | 124/233 (53%) | 109/233 (47%) |
| Non-COVID-19 (n=289)  Influenza A (n=82)  Influenza B (n=60)  Adenovirus (n=30)  Mycoplasma (n=20)  Cytomegalovirus (n=28)  Bacteria (n=48)  Fungus (n=21) | 50·0(47·0-53·0)  48·0(44·0-52·0)  62·0(57·0-68·0)  36·0(28·0-45·0)  18·0(8·0-28·0)  41·0(36·0-47·0)  60·0(55·0-65·0)  55·0(47·0-64·0) | 192/289 (67%)  49/82 (60%)  42/60 (70%)  25/30 (83%)  12/20 (60%)  18/28 (64%)  36/48 (75%)  10/21 (48%) | 97/289 (33%)  33/82 (40%)  18/60 (30%)  5/30 (17%)  8/20 (40%)  10/28 (36%)  12/48 (25%)  11/21 (52%) |

**Supplementary** **Table 7. Clinical characteristics of patients in the external validation cohort.**

**Extended Data 3. Additional Results on Model Development Set**

**Extended Data Figure 1. COVIDNet diagnostic performance on the internal test dataset.** **a,** Confusion matrix. **b,** ROC curve.

**b**

**a**

|  | **COVID-19 (n=151)** | **Non-COVID-19 (n=218)** |
| --- | --- | --- |
| Accuracy | 95.9% (93.8%-97.8%) | |
| AUC | 0.986 (0.971-0.996) | |
| Sensitivity | 92.0% (87.4%-96.1%) | 98.6% (96.8%-100.0%) |
| Specificity | 98.6% (96.8%-100.0%) | 92.0% (87.4%-96.1%) |
| PPV | 97.9% (95.1%-100.0%) | 94.7% (91.6%-97.4%) |
| NPV | 94.7% (91.6%-97.4%) | 97.9% (95.1%-100.0%) |
| F1 score | 95.0% (92.0%-97.3%) | 96.6% (94.8%-98.2%) |

**Supplementary** **Table 8. COVIDNet sensitivity analysis on the internal test dataset.** Model performance was evaluated on the first scan of the patients excluding the 17 patients. AUC=Area under the ROC curve. PPV=positive predictive value. NPV=negative predictive value.

**Extended Data Figure 2. COVIDNet sensitivity analysis on the internal test dataset**. **a,** Confusion matrix. **b,** ROC curve.

**b**

**a**

**Extended Data 4. Additional Results on External Validation Set**

|  | All radiologists | Radiologists A-D | Radiologists E-H |
| --- | --- | --- | --- |
| COVIDNet | 0.745 | 0.746 | 0.678 |
| Radiologists A-D | ·· | ·· | 0.853 |
| Radiologists working in the COVID-19 designated hospitals | | | |
|  | Radiologist B | Radiologist C | Radiologist D |
| Radiologist A | 0.697 | 0.720 | 0.679 |
| Radiologist B | ·· | 0.757 | 0.789 |
| Radiologist C | ·· | ·· | 0.763 |
| Radiologists not working in the COVID-19 designated hospitals | | | |
|  | Radiologist F | Radiologist G | Radiologist H |
| Radiologist E | 0.580 | 0.402 | 0.621 |
| Radiologist F | ·· | 0.636 | 0.518 |
| Radiologist G | ·· | ·· | 0.636 |

**Supplementary Table 9. Cohen’s κ.** Radiologists A-D are from the COVID-19 designated hospitals and Radiologists E-H are not from the COVID-19 designated hospitals. We used the majority vote to aggregate the classification results within either radiologist group. To be conservative, when a tie occurred, we assigned the case to the COVID-19 class as the collective decision.

**Extended Data 5. CT Manifestation Information**

|  | **probability of COVID-19** | **CT manifestations** |
| --- | --- | --- |
| G1 | 1.0000 | multiple GGO in subpleural area of both lungs |
| G2  G2a  G2b  G2c | 0.9993  0.9969  0.9938 | influenza B, extensive GGO, bulla  influenza B, extensive GGO, cavity  bacteria, extensive GGO |
| G3  G3a  G3b  G3c  G3d  G3e  G3f  G3g  G3h  G3i | 0.0000  0.0002  0.0015  0.0059  0.0005  0.0010  0.0038  0.0036  0.0052 | extensive consolidation  no definite finding  extensive GGO with partial consolidation  multiple GGO in both lungs  pleural effusion, interstitial edema, and partial consolidation  pleural effusion, interstitial edema, and partial consolidation  no definite finding  extensive GGO with partial consolidation  no definite finding |

**Supplementary Table 10. CT manifestations of the highlighted cases in the t-SNE visualization.**

**Extended Data 6. Application of COVIDNet**


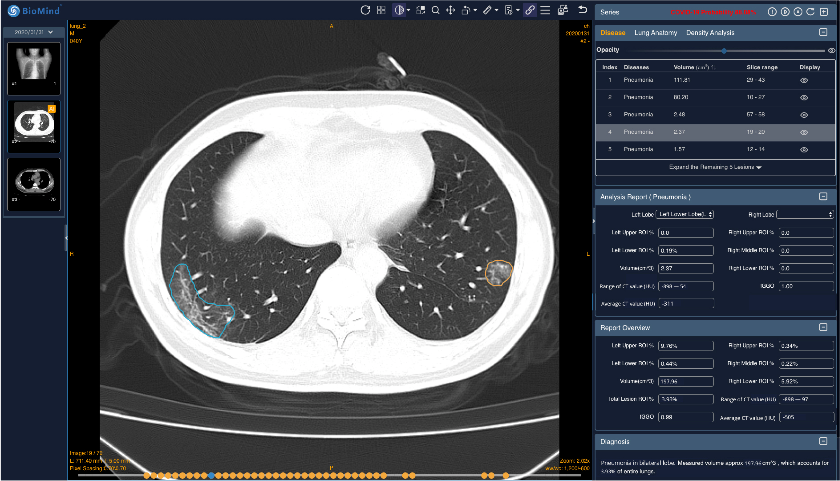


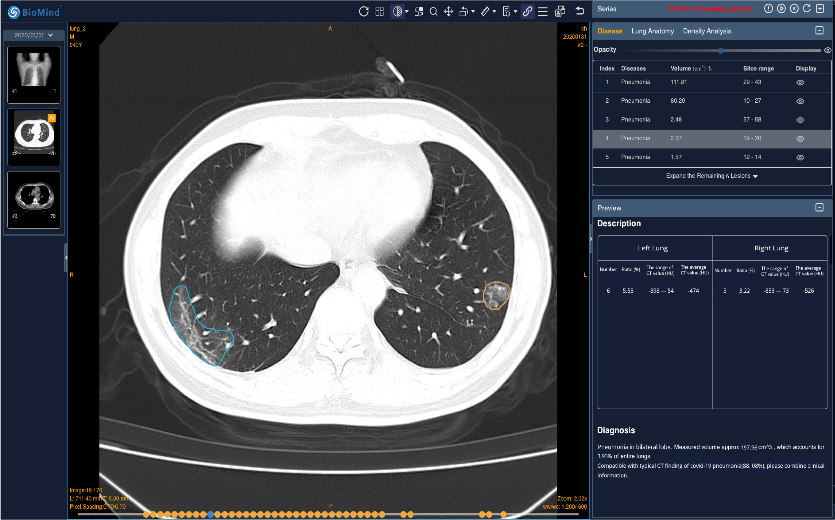


**Extended Data Figure 3. The user interface of COVIDNet.** The outline of the orange and blue lines indicated the appearance of a COVID-19 lesion in both lungs. Meanwhile, the COVID-19 probability was shown in red at the top right corner.

|  | **Numbers of CT scan** | **Sensitivity** | **Specificity** |
| --- | --- | --- | --- |
| **Total** | 11966 | 90.52% | 88.51% |
| **Luzhou People's Hospital** | 600 | 90.00% | 75.00% |
| **Wenzhou Central Hospital** | 849 | 100.00% | 89.26% |
| **Xiamen Fifth Hospital** | 125 | 96.00% | NA |
| **Hubei Cancer Hospital** | 10016 | 100.00% | 88.71% |
| **Union Hospital Affiliated with Tongji Medical College of Huazhong University of Science and Technology** | 198 | 95.45% | NA |
| **Zhongnan Hospital of Wuhan University** | 178 | 82.02% | NA |

**Supplementary Table 11. The sensitivity and specificity values of COVIDNet in the real-world application, related to Figure 4.** NA, not available.

**Extended Data 7. CT Device Information and the Protocol**

The CT scanner information is summarized as follows: (from Philips) Brilliance 6,16, and 64, Ingenuity Core 128, and Access CT, (from GE Medical Systems) BrightSpeed, Brivo CT385 Series, LightSpeed 16/ pro 16/ VCT, Optima CT520 Series/ CT540/ CT660/ CT680 Series, ProSpeed, Revolution ACT/ HD, (from Hitachi Medical Corporation) SupriaNMS NeuViz 64 In, (from Siemens) Emotion 16/ Duo/ Perspective/ Scope/ SOMATOM Definition AS/ AS+/ Flash/ go.Now/ go.Top/ Scope/ Spirit, (from TOSHIBA) Aquilion/ Aquilion PRIME, and (from UIH) uCT 510/ 530/ 760. The scans acquired protocol was presented below in Supplementary Table 9.

| **Scanning Parameters** | **Value** |
| --- | --- |
| Tube voltage | 70-120kV |
| Tube current | Automatic (40-250mA) |
| Noise index (NI) | 25 |
| Pitch | 0.984:1 |
| Matrix | 316×316, 512×512 |
| Slice collimation | (16-128) x (5-10mm)) |
| Slice interval  Recon. slices Thickness | 0 mm  0.5-1.25mm |
| Radiation dose | 1.5- 5.0mSv |

**Supplementary** **Table 12. The protocol of scan acquisition.**

**Extended Data Figure 4. CT device distribution in the training, internal test, and external test datasets.**

**Extended Data 8. Image Preprocessing**

| **Raw images** | **Preprocessed images** |
| --- | --- |
| **** | **** |
| **** | **** |
| **** | **** |

**Extended Data Figure 5. Raw CT images and the corresponding preprocessed scans.**

**Extended Data 9. Model Structure**

**Extended Data Figure 6. Structure of COVIDNet.**
